# Peripheral blood RNA gene expression profiling in patients with bacterial meningitis

**DOI:** 10.3389/fnins.2013.00033

**Published:** 2013-03-18

**Authors:** Margit Lill, Sulev Kõks, Ursel Soomets, Leonard C. Schalkwyk, Cathy Fernandes, Irja Lutsar, Pille Taba

**Affiliations:** ^1^Department of Neurology and Neurosurgery, University of TartuTartu, Estonia; ^2^Department of Physiology, University of TartuTartu, Estonia; ^3^Centre for Translational Research, University of TartuTartu, Estonia; ^4^Department of Biochemistry, University of TartuTartu, Estonia; ^5^Social, Genetic and Developmental Psychiatry Centre, Institute of Psychiatry, King's College LondonLondon, UK; ^6^Institute of Microbiology, University of TartuTartu, Estonia

**Keywords:** bacterial meningitis, gene expression profiling, gene networks

## Abstract

**Objectives:** The aim of present study was to find genetic pathways activated during infection with bacterial meningitis (BM) and potentially influencing the course of the infection using genome-wide RNA expression profiling combined with pathway analysis and functional annotation of the differential transcription.

**Methods:** We analyzed 21 patients with BM hospitalized in 2008. The control group consisted of 18 healthy subjects. The RNA was extracted from whole blood, globin mRNA was depleted and gene expression profiling was performed using GeneChip Human Gene 1.0 ST Arrays which can assess the transcription of 28,869 genes. Gene expression profile data were analyzed using Bioconductor packages and Bayesian modeling. Functional annotation of the enriched gene sets was used to define the altered genetic networks. We also analyzed whether gene expression profiles depend on the clinical course and outcome. In order to verify the microarray results, the expression levels of ten functionally relevant genes with high statistical significance (CD177, IL1R2, IL18R1, IL18RAP, OLFM4, TLR5, CPA3, FCER1A, IL5RA, and IL7R) were confirmed by quantitative real-time (qRT) PCR.

**Results:** There were 8569 genes displaying differential expression at a significance level of *p* < 0.05. Following False Discovery Rate (FDR) correction, a total of 5500 genes remained significant at a *p*-value of < 0.01. Quantitative RT-PCR confirmed the differential expression in 10 selected genes. Functional annotation and network analysis indicated that most of the genes were related to activation of humoral and cellular immune responses (enrichment score 43). Those changes were found in both adults and in children with BM compared to the healthy controls. The gene expression profiles did not significantly depend on the clinical outcome, but there was a strong influence of the specific type of pathogen underlying BM.

**Conclusion:** This study demonstrates that there is a very strong activation of immune response at the transcriptional level during BM and that the type of pathogen influences this transcriptional activation.

## Introduction

Despite the availability of effective antibiotics, health care and advances in adjunctive therapies, bacterial meningitis (BM) is still one of the top 10 causes of infection related mortality worldwide. Fatality rates as high as 20% for pneumococcal meningitis and 7% for meningococcal meningitis are reported. Among the survivors, 30–50% have permanent neurological sequelae (Weisfelt et al., 2006; Chaudhuri et al., 2008). Therefore, better understanding of the pathophysiological processes and the host's acute inflammatory response could ameliorate treatment and management of BM.

Bacterial invasion into the cerebrospinal fluid (CSF) induces a rapid inflammatory response, which is mediated by the innate immune system. Microglia and astrocytes are equipped with germline-encoded receptors, termed pattern-recognition receptors (PRRs), which have evolved to recognize highly conserved antigens, the so-called PAMPs (pathogen-associated molecular patterns) (Konat et al., 2006). PRRs include the family of Toll-like receptors (TLRs), of which TLR2 is the most important mediator of the inflammatory response to Gram-positive and TLR4 to Gram-negative infection including BM (Koedel et al., 2003; Konat et al., 2006; Neher and Brown, 2007). Many proinflammatory cytokines are released early into the circulation in response to sepsis or systemic inflammation. In cases of meningitis, cytokines, and adhesion molecules released by peripheral immune organs in the bloodstream cross the altered blood-CSF barrier and contribute to their CSF levels (Trojano et al., 1996; Fassbender et al., 1997).

The inflammatory response to bacterial invasion as a systemic reaction has been studied in clinical trials investigating sepsis on numerous occasions. The prevailing theory is that the host's response during sepsis, and likely also in BM, represents a pathological inflammatory response and therefore causes more damage than the pathogen itself (Bone et al., 1992; Stone, 1994; Warren, 1997; Hotchkiss and Karl, 2003; Deisenhammer et al., 2006). However, it is not known whether this pathological inflammatory response is triggered by transcriptional changes and whole genome expression profiling, to the best of our knowledge, has not been performed in BM patients to date.

The aim of our study was to identify up- or down-regulated transcriptional pathways during the acute phase of BM in RNA extracted from whole blood using whole genome transcriptional profiling combined with pathway analysis and functional annotation. In addition, we analyzed the possible factors (type of pathogen and outcome of the disease) associated with the gene expression profile in patients with BM.

## Methods

### Study participants

The study was conducted in the Tallinn Children's Hospital, the North Estonian Medical Centre, the Tartu University Hospital, the West Tallinn Central Hospital and the East Tallinn Central Hospital between the 1st of January and the 31st of December 2008 and included 21 patients with culture proven BM and 18 healthy controls. The healthy control group consisted of medical personnel and was free of any acute infection and had not taken any antibiotics within the month prior to blood sampling. BM was diagnosed if in addition to clinical signs of BM, there was a positive CSF culture of meningitis causing bacteria and/or positive blood culture in presence of CSF pleocytosis ≥10 × 10^6^ cells/L and/or positive CSF latex agglutination test with pleocytosis ≥500 × 10^6^ cells/L. On admission, all patients underwent a full clinical laboratory screening including a head computed tomography (CT). All microbiological analyses were performed according to the Clinical and Laboratory Standards Institute (CLSI) criteria in the local hospital laboratories.

The patients' outcome was evaluated by a neurologist at discharge from the hospital as favorable (normal, 13 patients) or poor outcome (with different complications, six patients), based on objective neurologic examination. Neurologic sequelae included cranial nerve disorders, paresis, ataxia, aphasia, cognitive impairment, apallic state, and epileptic seizures (Table 1). In two cases, the patients died following infection with BM.

**Table 1:** **Characteristics of the study population**.

**ID**	**Age**	**Previous antibiotics**	**Laboratory values**				**Causative organism**	**Outcome**
			**Blood WBC (10^9^ cells/L)**	**CRP(mg/L)**	**CSF WBC (10^9^ cells/L)**	**CSF Protein g/L**		
M028	22 days	No	8.7	147	13.4	2.1	*S. agalactiae*	Epileptic syndrome
M039	17 days	No	4.1	87	5.1	2.5	*E.coli*	Normal
M031	55 years	No	8.1	219	1.5	10.1	*S. pneumoniae*	Died
M032	75 years	Yes	21.8	251	7.7	1.7	*S. pneumoniae*	Spastic tetraparesis
M027	52 years	No	32.2	77	0.3	7.5	*S. pneumoniae*	Aphasia, right spastic hemiparesis
M022	54 years	No	9.6	171	1.2	2.6	*S. pneumoniae*	Normal
M041	46 years	No	26.3	5	0.07	0.5	*S. pneumoniae*	Died
M046	13 years	No	18.6	109	4.6	2.3	*S. pneumoniae*	Normal
M040	73 years	No	18.4	231	1.8	1.8	*L. monocytogenes*	Normal
M029	37 years	Yes	24.2	197	2.3	2.0	*S. pneumoniae*	Normal
M004	57 years	No	13.4	63	4.5	2.7	*H. influenzae*	Normal
M014	58 years	No	9.7	54	2.3	9.8	*S. pneumoniae*	Cognitive dysfunction, anisocoria, left spastic hemiparesis
M023	70 years	Yes	11.4	174	4.3	6.6	*S. pneumoniae*	Normal
M047	75 years	No	34.6	146	0.04	0.6	*S. pneumoniae*	Cognitive dysfunction
M036	17days	No	2	105	0.2	6.9	*S. agalactiae*	Normal
M042	71 years	No	20.4	421	0.3	1.5	*S. pneumoniae*	Normal
M025	47 years	No	5.8	285	1.1	0.4	*N. meningitidis C*	Facial nerve paresis, ataxia
M012	6 years	No	4.3	19	0.06	2.9	*S. pneumoniae*	Normal
M002	7 years	No	21.9	109	1.7	4.6	*N. meningitidis*	Normal
M006	55 years	No	11.2	321	3.4	5.8	*S. pneumoniae*	Normal
M038	60 years	No	16.2	15	0.7	7.4	*S. pneumoniae*	Normal

Additional comparisons were made in order to assess whether there was a relationship between gene expression profiles and the specific pathogens mediating BM and/or the clinical outcome in the patients. Comparisons were made between three pathogen groups—(A1) pneumococci, (A2) other bacteria and (A3) controls. Clinical outcome was divided into (B1) favorable outcome without sequelae, (B2) poor outcome with neurologic sequelae or death and (B3) healthy controls.

### Sample collection and RNA preparation

For RNA expression analysis, 6 ml blood was collected via venipuncture into Tempus tubes (Applied Biosystems, Foster City, USA) within 48 h of admission, and stored first at −20°C for a maximum of 3–4 months, then transported in dry ice. After thawing, RNA was extracted from whole blood according to the manufacturer's protocol (Applied Biosystems PN 4379228C). Alpha and beta globin mRNA was depleted with the GlobinClear Whole Blood Globin Reduction kit (Ambion, Austin, USA). The quality of RNA was checked with a Bioanalzer 2100 (Agilent, Santa Clara, USA) and gene expression profiling was performed with GeneChip Human Gene 1.0 ST Arrays (Affymetrix, Santa Clara, USA), which can measure the transcription of 28,869 genes.

### Microarray hybridization and analysis

The RNA was labeled using the Affymetrix GeneChip Whole Transcript (WT) Sense Target Labeling Assay (Affymetrix, Santa Clara, USA). This assay is designed to generate amplified and biotinylated sense-strand targets from the entire expressed genome without bias. Briefly, double-stranded complementary DNA (cDNA) was synthesized from 300 ng of total RNA by reverse transcription using random hexamers tagged with a T7 promotor primer sequence. The double-stranded cDNA was subsequently used as a template and amplified by T7 RNA polymerase producing several copies of antisense *complementary RNA* (cRNA). In the second cycle of cDNA synthesis, random hexamers were used to prime reverse transcription of the cRNA from the first cycle to produce single-stranded DNA in the sense orientation. This DNA was fragmented with a combination of uracil DNA glycosylase (UDG) and apurinic/apyrimidinic endonuclease 1 (APE 1). DNA was labeled by terminal deoxynucleotidyl transferase (TdT) and hybridization was performed according to the manufacturer's protocol. The arrays were subsequently washed, stained with phycoerythrin streptavidin and scanned according to standard Affymetrix protocols. Images were processed using the Affymetrix Microarray Suite 5.0 Expression Console and image quality subsequently assessed. The processed data files were further analyzed using Bioconductor *affy* and *limma* packages. Gene expression data (.cel files) and study design information has been uploaded to the public database Gene Expression Omnibus (accession number GSE40586).

### Quantitative real-time PCR (qRT-PCR) analysis

In order to verify the microarray results, genes from the gene expression profiling were sorted according to the degree of statistical significance of the differential expression. Ten genes with the lowest *p*-values were selected and further analyzed with qRT-PCR: CD177, IL-1R2, IL-18R1, IL-18RAP, OLFM4, TLR5, CPA3, FCER1A, IL5RA, and IL7R. RNA was converted into cDNA using High Capacity cDNA Synthesis kit from Applied Biosystems (4368814). TaqMan assays and Gene Expression Master mix was used for the qRT-PCR reaction generated in the SDS 7900 HT system (Applied Biosystems, CA, USA).

### Functional annotation of differentially expressed genes

The Functional Analysis of a gene network is used to identify the biological functions that are most significantly related to the molecules in the network. To define the functional networks of differentially expressed genes, data was analyzed by using the Ingenuity Pathway Analysis (IPA, Ingenuity Systems, *www.ingenuity.com*) that calculates a significance score (network score) for each network. This score indicates whether the likelihood that the assembly of a set of focus genes in a network could be explained by random chance alone (e.g., score of 2 indicates that there is a chance of 1 in 100 that the focus genes are together in a network due to random chance). A data set containing the Affymetrix probeset identifiers and their corresponding fold change (log2) values were uploaded into the IPA software. Each gene identifier was mapped to its corresponding gene object in the Ingenuity Pathways Knowledge Base to identify molecules whose expression was significantly differentially regulated (focus genes or Networks Eligible molecules). These focus genes were overlaid onto a global molecular network developed from information contained in the Ingenuity Knowledge Base. Networks of these focus genes were then algorithmically generated based on their connectivity.

A network is a graphical representation of the molecular relationships between genes or gene products, which are represented as nodes, and the biological relationship between two nodes is represented as an edge (line). All edges are supported by at least one reference from the literature, or from canonical information stored in the Ingenuity Pathways Knowledge Base.

### Statistical analysis

The normalized, background subtracted and modeled expression (Robust Microarray Analysis, RMA) data was further analyzed using Bayesian model moderated *t*-test implemented in the Bioconductor *limma* package of the statistical software R (http://www.r-project.org/) (Smyth, 2004). False Discovery Rate (FDR) was used to adjust *p*-values and to correct for the multiple testing issues (Storey and Tibshirani, 2003). Comparisons between the groups by pathogens and clinical outcome were performed, using general linear models of analysis of variance (ANOVA), to test the effect of these factors on gene expression pattern. Sample comparisions for the qRT-PCR reaction data were made using Welch's *t*-test.

The Ethics Review Committee on Human Research of the University of Tartu approved the study. All subjects or their legal representatives signed the informed consent.

## Results

The details of study patients are presented in Table 1. There were 21 patients (median age 54 years, IQR 13, 60; 12 males), and 18 healthy controls (median age 54 years, IQR 47, 60; 5 males). Two patients died, neurological sequelae were observed in six patients, and the remaining 13 patients had a normal outcome. Altogether 14 patients had a pneumococcal infection, two patients had a group B streptococcus (GBS) infection, and another two patients had meningococcal meningitis. *Escherichia coli*, *Haemophilus influenzae*, and *Listeria monocytogenes* were found in one patient.

### Genome-wide expression profiling

Comparison of the blood RNA samples isolated from BM patients and healthy controls revealed distinct gene expression profiles. Altogether 5500 genes out of the analyzed 28,869 genes showed statistically significant differential expression at the FDR adjusted p-values ≤0.01. Relative differences in the expression signal (fold change or logFC) between these two groups were of moderate effect size. In the BM patients, 47 genes were up-regulated more than 1.5 fold and 93 down-regulated more than 1.5 fold, compared to controls. In addition, the high B-statistics values for the list of genes were indicative of genuine biological differences between these groups (Table 2). As it appears from the gene annotations, most of these genes were related to immune regulation and the anaphylactic response (e.g., FCER1A, CPA3, MS4A2; Tables A1, A2).

**Table 2:** **Twenty most significantly up- or down-regulated genes**.

**Probeset**	**Sym**	**LogFC**	**AveExpr**	***T***	***P*-value**	**Adjusted *P*-value**	***B*-value**	**Gene name**
7906443	FCER1A	2.48	6.42	12.04	4.51E-15	1.46E-10	23.77	Receptor for Fc fragment of IgE, alpha polypeptide
8083260	CPA3	1.66	5.35	10.24	6.77E-13	1.09E-08	19.08	Carboxypeptidase A3 (mast cell)
7940226	MS4A2 (FCER1B)	1.19	4.94	9.62	4.22E-12	3.12E-08	17.35	Receptor for Fc fragment of IgE, beta polypeptide
7971950	DACH1	−1.54	7.03	−9.58	4.81E-12	3.12E-08	17.23	Dachshund homolog 1
8048847	HRB	−1.51	11.13	−9.49	6.30E-12	3.12E-08	16.97	HIV-1 Rev binding protein (AGFG1)
7988672	HDC	1.35	6.26	9.47	6.68E-12	3.12E-08	16.92	Histidine decarboxylase
8139656	GRB10	−2.33	7.87	−9.44	7.35E-12	3.12E-08	16.83	Growth factor receptor-bound protein 10
8151512	PAG1	−1.17	9.83	−9.42	7.73E-12	3.12E-08	16.78	Csk-binding protein
8103094	NR3C2	1.09	6.07	9.28	1.19E-11	4.27E-08	16.37	Mineralocorticoid receptor
8129618	VNN1	−2.98	10.04	−9.15	1.77E-11	5.33E-08	15.99	Vanin 1
8169249	MID2	0.95	5.71	9.14	1.82E-11	5.33E-08	15.97	Midline 2
7956878	IRAK3	−1.98	10.44	−9.09	2.11E-11	5.62E-08	15.82	Interleukin-1 receptor-associated kinase 3
8106626	NA	−1.30	7.04	−9.07	2.26E-11	5.62E-08	15.76	
8169154	NA	−1.33	6.29	−9.01	2.68E-11	5.95E-08	15.60	
8105579	IPO11	−1.58	5.35	−9.00	2.76E-11	5.95E-08	15.57	Importin 11
7980381	TMED8	−1.25	8.43	−8.98	2.95E-11	5.96E-08	15.51	Transmembrane emp24 protein
8112896	ANKRD34B	−2.12	6.34	−8.83	4.75E-11	9.03E-08	15.05	Ankyrin repeat domain 34B
8103399	PDGFC	−1.52	7.69	−8.77	5.70E-11	1.02E-07	14.88	Platelet derived growth factor C
8044035	IL18R1	−2.97	8.71	−8.73	6.37E-11	1.03E-07	14.77	Interleukin 18 receptor 1

### Gene expression pattern BM vs. controls

**Figure 1:**
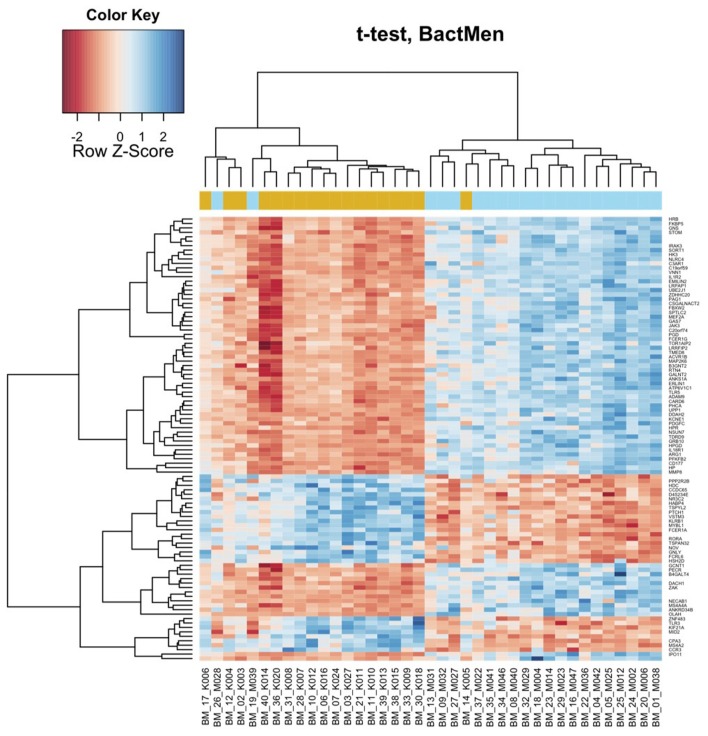
**Bacterial meningitis patients have a distinct profile on the heatmap of the blood derived gene expression levels.** The top 100 genes (vertical dimension) from the decreasing ordered list of moderated *t*-values were clustered according to the distances between their gene expression values. Signals are scaled to *Z*-scores of the rows. The colored bar above the heatmap (horizontal dimension) indicates the grouping variable—golden rod for meningitis patients (M), blue for healthy controls (K).

**Figure 2:**
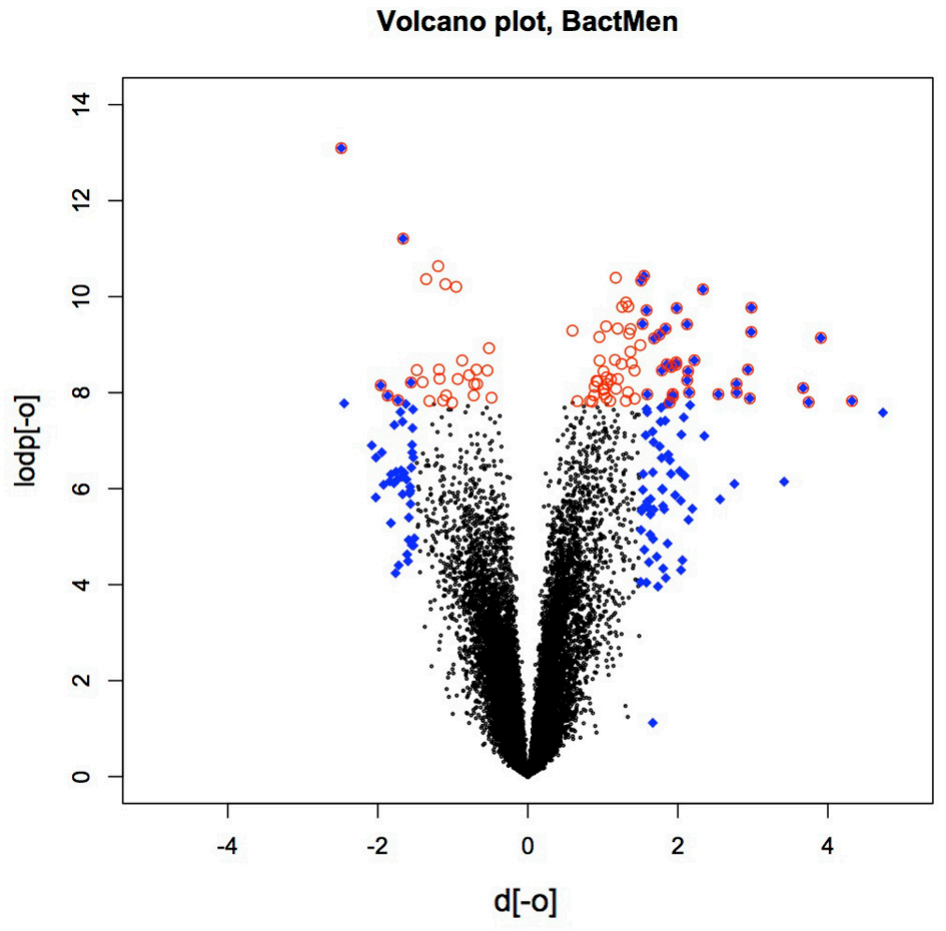
**The volcano plot depicting the fold differences in gene expression levels between the bacterial meningitis patients and healthy controls.** Colored points refer to the top 100 transcripts according to fold change (blue 612 diamonds) and *p*-value (red circles).

Heatmap (Figure 1) and a volcano plot (Figure 2) illustrate the general gene expression pattern in relation to the main factor—diagnosis of BM. The heatmap indicates a good clustering of samples according to whether the infection was present or absent. There is a clear distinction between these two groups and the gene expression profiles were able to discriminate between the two main groups (Figure 1). The volcano plot illustrates a high number of statistically significant differences (*p* < 10e-06 is the equivalent for Bonferroni corrected *p* < 0.05) (Figure 2). Moreover, the ratio of the differential expression (fold change, illustrated in the abscissa of the volcano plot) is also quite remarkable. Therefore, there was very good correlation between the fold change differences and p-values (i.e., genes with a large fold change difference also had a low *p*-value in the group-wise comparison). Two vertical axes denote the position of a 1.5 and −1.5 fold change difference in the gene expression profiles between disease cases and controls (Figure 2).

In Table 1, the patients are lined up the same order as in the heatmap (Figure 1). With two exceptions, the BM patients and controls are clearly separated as they have very distinct gene expression profiles. The two exceptions clustering within controls (M028 and M039) were both neonates one with *E. coli* and the other with GBS meningitis.

Additional statistical modeling was performed to assess whether the type of pathogen or the outcome of the disease influenced the gene expression profiles measure in the patient group. Two separate linear models in which the gene expression differences were analyzed for the general effect of clinical outcome [R code: design ← model.matrix (~0 + eset$outcome)] or for the general effect of pathogen [R code: design ← model.matrix (~0 + eset$bacteria)]. Subjects were allocated to one of three pathogen groups: infected with *S. pneumoniae;* infected with other pathogens; controls (non-infected). After general modeling pair-wise comparisons between groups were performed. The type of pathogen significantly influenced the expression profile. Table 3 (additional information in Table A3) illustrates gene expression differences if *S. pneumoniae* or other pathogens cause BM. The gene expression profile was significantly different in patients with pneumococcal meningitis compared to BM caused by other pathogens. However, the genes listed in Table 3 differ from the genes responsible for the general effect of BM (BM patients versus controls, see Table 2). We have to consider the pathogen heterogeneity here. While the number of patients with *S*. is 14, group with “other” pathogens is smaller (seven patients) and very heterogeneous (almost all pathogens as single entries). Therefore, this comparison is exploratory without any conclusive outcome.

**Table 3:** **The type of pathogen caused differences in gene expression profiles and table illustrates comparison between two groups of BM patients, *S. pneumonia* vs. other pathogens**.

**Probeset**	**Gene**	**LogFC**	**AveExpr**	***T***	***P*-value**	**Adjusted *P*-value**	***B*-value**	**Gene name**
7893004	NA	2.20	4.68	5.84	7.71E-07	0.025	5.399	
7932109	SEPHS1	0.64	7.94	5.45	2.71E-06	0.044	4.325	selenophosphate synthetase 1
7909455	TRAF3IP3	0.76	9.28	5.02	1.09E-05	0.085	3.136	TRAF3 interacting protein 3
8006634	PIGW	0.85	5.63	4.96	1.32E-05	0.085	2.966	phosphatidylinositol glycan class W
7945071	FOXRED1	0.34	6.64	4.89	1.66E-05	0.085	2.770	FAD-dependent oxidoreductase
8103867	WWC2-AS2	−0.43	5.31	−4.85	1.89E-05	0.085	2.660	WWC2 antisense RNA 2
7948894	RNU2-1	−2.29	6.85	−4.81	2.11E-05	0.085	2.563	RNA. U2 small nuclear 1
7980309	C14orf1	0.66	7.73	4.70	3.04E-05	0.085	2.251	probable ergosterol biosynthetic protein 28
8173269	LAS1L	0.63	7.04	4.63	3.83E-05	0.085	2.052	LAS1-like
8088458	FHIT	0.59	6.71	4.59	4.32E-05	0.085	1.947	fragile histidine triad gene
8008517	NME1	0.88	6.77	4.58	4.50E-05	0.085	1.914	NME/NM23 nucleoside diphosphate kinase 1
7915718	TESK2	−0.57	7.90	−4.57	4.54E-05	0.085	1.905	testis-specific kinase 2
7916020	NA	−0.50	3.38	−4.57	4.55E-05	0.085	1.904	
8020691	PSMA8	0.38	3.89	4.57	4.64E-05	0.085	1.887	Proteasome subunit type alpha 8
8119492	BYSL	0.51	6.88	4.54	5.05E-05	0.085	1.813	bystin-like
7899253	ZDHHC18	−0.80	10.42	−4.53	5.11E-05	0.085	1.803	zinc finger. DHHC-type containing 18
8094719	N4BP2	1.09	6.73	4.53	5.18E-05	0.085	1.791	NEDD4 binding protein 2
7892556	NA	0.76	12.28	4.52	5.39E-05	0.085	1.758	
7894790	NA	0.66	9.69	4.51	5.48E-05	0.085	1.743	
7978132	IPO4	0.46	7.22	4.47	6.29E-05	0.085	1.624	importin 4
7972548	GPR18	1.07	6.55	4.46	6.46E-05	0.085	1.601	G protein-coupled receptor 18
7927876	TET1	0.64	5.19	4.45	6.63E-05	0.085	1.579	tet oncogene 1
7930148	SFXN2	0.68	6.64	4.44	6.78E-05	0.085	1.560	sideroflexin 2
7903920	CHI3L2	1.30	6.05	4.44	6.81E-05	0.085	1.556	chitinase 3-like 2
8058670	IKZF2	1.27	6.10	4.44	6.86E-05	0.085	1.549	IKAROS family zinc finger 2

The clinical outcome was only modestly associated with gene expression profiles, and there were no statistically significant differences (*p* < 0.05 after FDR correction) between groups with a favorable outcome of BM and those with a poor outcome. However, analysis of the separate patient groups with favorable or poor outcome in comparison with healthy controls resulted in significant differences in gene expression profiles that probably reflect the effect of an acute infection.

A functional annotation of expression profiles was subsequently applied in order to identify functional changes in the context of genetic networks. Using IPA software, the dataset containing 5500 genes that had significant differential expression between BM and control groups was uploaded. The genetic network with the highest score (43) was related to antigen presentation, cell-mediated and humoral immune response (Table 4). Again, almost all activated networks were related to the immune response.

**Table 4:** **Top 10 most significantly activated pathways**.

**ID**	**Molecules in network**	**Score**	**Focus molecules**	**Top functions**
1	AIM2, B3GNT5, BPI, C4, C3AR1, CARD6, CD27, CD63, CD74, CEACAM8, CLEC2D (includes EG:29121), CRISP3 (includes EG:10321), CST7, EMR1, ETS, HLA-DMA, HLA-DMB, HLA-DPA1, HLA-DPB1, HLA-DQA1, HLA-DRA, IFN Beta, IL12, IL32, IL18R1, IL18RAP, KLRB1, MHC Class II, MHC II, MHC II-and beta, MHC2 Alpha, MYBL1, NFκB, PTGDR, and SOCS3.	43	26	Antigen presentation, cell-mediated immune response, and humoral immune response.
2	BMX, CCR4, CD2, CD3, CD4, CD5, CD6, CD8, CD28, CD52, CD247, CD3D, CD3E, CD3G, ERK, Fcer1, FCER1A, HDC, HSH2D (includes EG:84941), ICOS, IgE, ITK, MERTK, MS4A2, NFATcomplex, PAG1, PDGFC, PLCgamma, PLCG1, RASGRP1, TCR, TEC/BTK/ITK/TXK/BMX, TXK, VAV, and ZAP70.	40	25	Cell signaling, molecular transport, and vitamin and mineral metabolism.
3	ARG1, ARHGAP29, Calpain, CCL5, CCR7, CD3-TCR, CD40LG, CLC, CYP1B1, FGD4, Filamin, FLOT1, GNLY, GZMB, HPR, IgG, IgM, Integrin and alpha, ITGA4, ITGA7, ITGAM, ITGB7, Jnk, KLHL2, LRG1, MAP2K1/2, MMP, MMP8, MTF1, POR, PRF1, Rap1, Ras homolog, SAMSN1, and Tgf beta.	36	23	Cell-to-cell signaling and interaction, hematological system development and function, and immune cell trafficking.
4	ADM, Alcohol group acceptor phosphotransferase, BCL2A1, Calcineurin protein(s), CDC25B, Cpla2, Creb, CX3CR1, DPP4, GBA, GPR183, GZMA, hCG, HGF, HMGB2, Hsp27, IL1RL1, LDHA, LDHB, LDL, MAP2K6, MAPK14, Mek, MKK3/6, MKNK1, MS4A1, NFATC2, P38 MAPK, PRKCH, PRKCQ, R PS6KA5, Rsk, TNFAIP6, Vegf, and ZAK.	35	23	Cellular compromise, inflammatory disease, and genetic disorder.
5	AIM2, ANKS1A, ARG1, ARG2, Arginase, BTN3A1, BTN3A2, CD163, CLEC4E, CLEC5A, CYSLTR1, CYSLTR2, EIF1AY, GAS7, HLX, IFI44, IFI44L, IFNA2, IFNG, IGF1, IL13, IL13RA2, IL18R1, IL18RAP, IL4R, ISL1, LCN2, MS4A4A, P2RY5, PDLIM2, PECR, SAMSN1, SLC1A3, TRPS1, and UBD.	33	22	Antigen presentation, cell-mediated immune response, and humoral immune response.
6	Akt, CD163, CR1, FLT3, GC-GCR dimer, GRB10, Ifn gamma, IKK, IL1, IL-1R, IL-1R/TLR, IL1R1, IL1R2, IL2RB, IL5RA, IL7R, Interferon alpha, IRAK3, IRF, JUN/JUNB/JUND, KCNA3, LCN2, NEDD4, NF-and kappa; B, ORM1, Ptk, RARRES3, RNF125, SIGLEC5, STAT5a/b, TGFBR3, TIFA, Tlr, TLR2, and TLR5.	31	21	Cell-mediated immune response, hematological system development and function, and tissue morphology.
7	ACSL1, Actin, AMPK, CASP5, Caspase, CCNA1, CCND2, Cyclin A, Cytochrome c, DACH1, E2f, ERK1/2, Erm, GZMK, Histone h3, Hsp70, Hsp90, IFIT1, Insulin, KCNMA1, KLRG1, MME, MMP9, NFKBIA, NLRC4, NR3C2, PFKFB3, PI3K, PPARG, RNA polymerase II, SPN, STAT, TNFAIP3, TRAT1, and VNN1.	29	20	Cardiovascular disease, hematological disease, and neurological disease.
8	Ap1, ARL4C, B4GALT4, B4GALT5, CAMK4, CaMKII, CCR3, CEACAM1, CHI3L1, Coup-Tf, ETS1, ETS2, G alphai, GADD45A, Galactosyltransferase beta 1,4, HPGD, HRH4, LRRN3, LTF, Mapk, Myosin, Nfat, PADI4, Pak, Pdgf, PDGF BB, PFKFB2, PLEKHA1, PP2A, Rac, Rxr, SERPINB1, THBS1, VitaminD3-VDR-RXR, and WDFY3.	26	20	Gene expression, cardiovascular system development and function, and cellular movement.
9	24,25-dihydroxyvitamin D3, ACVR1B, ADCYAP1R1, ALP, ALPL, ASPH, ATP9A, CA4, Calmodulin, CD84, CD244, Ck2, CYP19A1, CYP2D6, DDAH2, F Actin, FSH, GPR56, LY9, Pka, Pkc(s), PLC, PRKACB, PRKACG, Proteasome, Ras, SH2D1A, SH2D1B, SH3BP4, SLAMF6, SLAMF7, SMPDL3A, STAT4, TCF7, and UPP1.	23	17	Cell death, cancer, and tumor morphology.
10	ATP13A3 (includes EG:79572), CALML3, CCNG2, CCR7, CD59, CD177, CD274, CEP170, CSF3, ENTPD7, GALNT14, GYG1, IDI1, IL17F, IL1RAPL1, LRRFIP2, MS4A5, MUC2, MUC13, MUC5AC, MYO10, NCR3, NR1D1, NR1D2, PCOLCE2, PIGA, PLEKHA6, PRTN3, RORA, SH3BGRL2, SORL1, TGFB1, TMED8, UBE2J1, and ZFP36.	20	15	Hematological disease, cell death, and immunological disease.

**Figure 3:**
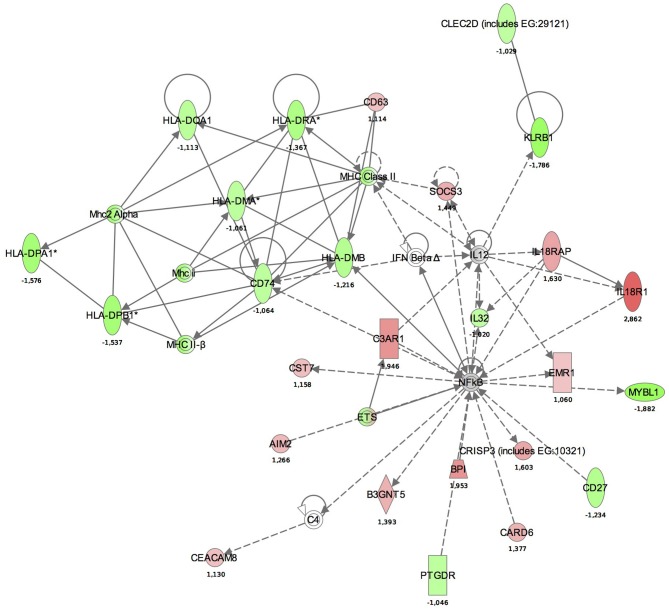
**Functional analysis of the gene expression networks, performed using Ingenuity Pathway Analysis (IPA).** This figure illustrates the activation of the antigen presentation pathway. The genes in green were down-regulated and the genes in red were up-regulated with respect to the healthy control group. (Numbers are fold changes).

**Figure 4:**
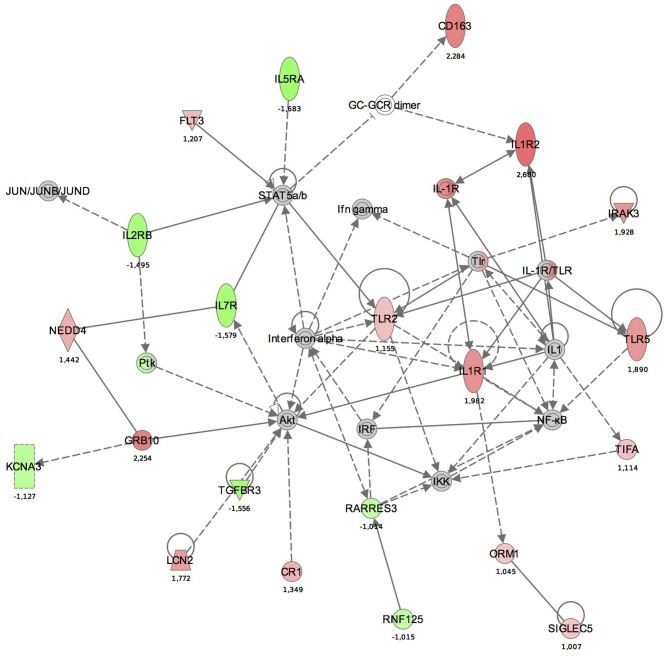
**The functional analysis of the gene expression networks, performed using Ingenuity Pathway Analysis (IPA).** This figure illustrates the activation of the cell-mediated immune response network. Genes in green were down-regulated and the in red were up-regulated with respect to the healthy control group. (Numbers are fold changes).

Maps for the two most significantly disturbed pathways, the antigen presentation and cellular immune response pathways were generated (Figures 3, 4). In the antigen presentation pathway, the up-regulated genes were the major histocompatibility complex (MHC) class II region encoded HLA subgroup genes—HLA-DR, HLA-DQ, HLA-DP. In the case of the cell-mediated immune response pathway, the up-regulated genes were associated with interlekins: interleukin-2 receptor subunit beta (IL2RB), interleukin-5 receptor alpha (IL5RA), interleukin-7 receptor (IL7R).

### Quantitative RT-PCR

**Figure 5:**
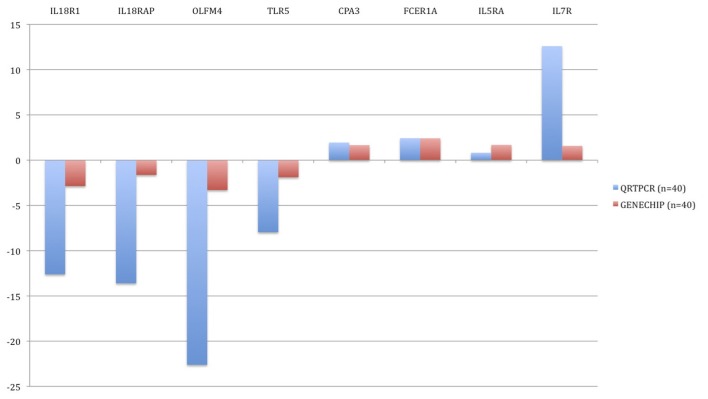
**Validation of microarray results by quantitative real-time PCR (qRT-PCR).** Blue bars represent the qRT-PCR data (relative expression, ΔCt) and the red bars are data from the microarray experiment. The data from the microarrays were strongly correlated with the qRT-PCR data.

In order to verify changes found in the microarray experiment, qRT-PCR was performed. Genes were sorted according to the statistical significance of their differential expression and 10 genes with the lowest *p*-values were selected. The following genes were analyzed: CD177, FCER1A, CPA3, IL1R2, IL18R1, IL18RAP, OLFM4, TLR5, IL5RA, and IL7R. In all 10 cases, qRT-PCR confirmed statistically significant changes in gene expression between the groups (Figure 5). In BM patients, CPA3, FCER1A, IL5RA, and IL7R were up-regulated and IL18R1, IL18RAP, OLFM4, TLR5, CD177, and IL1R2 were down-regulated.

## Discussion

The significant role of the immune response in determining the outcome of BM has been well characterized in the literature. The present exploratory study on gene profiling in patients with BM demonstrates that activation of the host's immune response occurs at the level of the transcriptome. Most of the up-regulated genes are derived from mast cells suggesting hyper-activation of the immune system and allergic response (Saito, 2008; Manikandan et al., 2012). However, there are still many significantly up- or down-regulated genes which have not been clearly associated with bacterial infections to date. As no other genome-wide gene expression profiling studies have been done in BM patients, we compared our results with earlier studies conducted on sepsis patients (Stone, 1994; Warren, 1997; Hotchkiss and Karl, 2003; Nduka and Parrillo, 2009).

In the early phase of sepsis, a full-blown activation of immune responses due to the release of high levels of damage associated molecular patterns (DAMPs) from invading microorganisms or damaged host tissue has been described. This leads to up-regulation of TLR expression (Nduka and Parrillo, 2009). Activation of TLR2 and TLR4 are some of the first steps in the immune response to BM, triggering the inflammatory cascade by the cytokines (Coimbra et al., 2006; Manikandan et al., 2012). Our findings on a systemic response to infection are quite general as the group of BM patients in our study was quite heterogeneous, with a wide range of ages and infecting organisms. As a general or common pattern for the BM, we found a significant activation of inflammatory and immune response genes. Common or general patterns in mammalian cell transcriptome response to pathogens causing meningitis have been described (Schubert-Unkmeir et al., 2009). Immune activation with up-regulation of interleukins and interferons has been accepted as a general response to meningitis (Schubert-Unkmeir et al., 2009). However, the results in literature and the design of these experiments are quite different. While several studies have described activation of different components of the immune response, other studies have used animal models or *ex vivo* blood samples, no systemic whole transcriptome analysis of blood samples from BM patients has been performed so far (Pathan et al., 2004; Schubert-Unkmeir et al., 2009; Del Tordello et al., 2012). Therefore, our study describes a unique approach, analyzing blood samples of BM patients for whole genome transcription profiling.

In addition to the general pattern, we found some pathogen specific footprints in the gene expression profile in single cases. Cluster analysis of these gene expression profiles demonstrated that the only case of *E. coli* meningitis in the 17 days old baby (BM_19_M039) clustered within the control group (Figure 1). Whether this is due to the fact that the blood gene expression profile is pathogen specific or because this case occurred in a neonate, or both, remains to be proven in larger studies. Another case of neonatal meningitis clustering among the controls had a new episode of BM few weeks later. This may have been indicative of an abnormal immune response in that child. Previous antimicrobial therapy was not associated with clustering. Both patients (BM_19_M039 and BM_26_M028) were children with an age less than one month but the age cannot be the only reason for different clustering as the third, very young patient did not show a similar gene expression profile to BM_19_M039 and BM_26_M028. Differential clustering was also supported in the analysis using a linear model, where a significant effect of pathogen type was found on the differential expression.

All the samples were collected from the patients during hospitalization, during the acute phase of the disease. Therefore, expression of the genes related to pathogen identification and pro- and anti-inflammatory cellular signaling was expected to be increased (Coimbra et al., 2006). In addition to common immune system activation genes (interleukins etc.), we were able to see quite specific hypersensitivity-related gene expression pattern, not described for BM so far. Indeed, the three most up-regulated genes were FCER1A, CPA3, and MS4A2, all closely related to the early immune response and hypersensitivity. FCER1A is involved in inducing the inflammatory and immediate hypersensitivity responses in allergic disorders such as hay fever, asthma, and chronic idiopathic urticaria (Bleehen et al., 1987; Bruhn, 2011; Chandrashekhar et al., 2011; Rueff et al., 2011). FCER1A seems to play a role in several pathways involved in both innate and adaptive immune responses (Van Vugt et al., 1996, 1999; Ernst et al., 1998; Edberg et al., 1999). CPA3, mast cell carboxypeptidase, is a distinctive carboxypeptidase which is exclusively located in mast cells and possesses pancreatic carboxypeptidase A (CPA)-like activity (Goldstein et al., 1989; Natsuaki et al., 1992). MS4A2 (FCER1B) plays a role in allergic or autoimmune diseases, such like Wegener's granulomatosis, asthma and atopic dermatitis (Grammatikos, 2008; Mathias et al., 2009; Sanak et al., 2009). Therefore, the list of up-regulated genes suggests a strong activation of the anaphylactic response genes and mast cells in addition to the immune system activation. This finding could explain the complicated clinical course of BM. Immune response factors can be deleterious to the functions of an organism. Interleukin 6 has been shown to cause myocardial dysfunction during meningococcal septic shock (Pathan et al., 2004). Interestingly, activation of mast cells is not necessary deleterious as mast cells can increase survival in certain sepsis models (Piliponsky et al., 2008).

Similar to studies on systemic response (Schubert-Unkmeir et al., 2009; Echenique-Rivera et al., 2011), we also observed networks with functions including “Antigen presentation,” “Cell signaling,” “Cell-mediated immune response,” and “Humoral immune response” among the top activated pathways. In the antigen presentation pathway, the up-regulated genes were MHC class II region encoded HLA subgroup genes—HLA-DR, -DQ, -DP. The MHC-II locus consists of a group of 7–10 highly polymorphic genes that code for the alpha and beta chains of the classical MHC-II heterodimeric molecules. These molecules function by presenting antigenic peptides to CD4+ T lymphocytes and are critical in the development of the T cell repertoire, proliferation and differentiation of antigen-specific CD4+ T cells during adaptive immune responses. HLA-DM, also present in our pathway aids this process, as it is a MHC class-II-associated molecule (Majumder et al., 2006).

In the antigen presentation pathway, down-regulated genes were IL18R1 and IL18RAP. IL18R is a key regulator of TH1 cells. Binding of IL-18 to IL-18R stimulates TH1 but also TH2-type cytokine release depending on its cytokine milieu. A strong association between a single-nucleotide polymorphism (SNP) located in IL18R1 and asthma and atopic phenotypes has been recently observed (Reijmerink et al., 2008).

In the cell-mediated immune response pathway, the following genes were up-regulated: IL2RB, IL5RA, IL7R. The IL2RB protein is expressed in large granular lymphocytes (LGL) in their resting state. This beta subunit is involved in receptor mediated endocytosis and transduces the mitogenic signals of IL2 (http://www.genecards.org). Interleukin 5 receptor alpha (IL5RA) is an IL5 receptor and binds to IL5. Anti-inflammatory cytokines, including IL10, IL4, IL13, and IL5, are synthesized from Th2 immune cells. It has been found that increased expression of IL5RA on CD34+ cells favors eosinophilopoesis and therefore may contribute to the subsequent development of blood and tissue eosinophilia, a hallmark of allergic inflammation (Sehmi et al., 1997).

Some limitations of the study should be noted. Due to the rarity of BM, the patients group is very small and heterogeneous in terms of age and infecting organisms. Heterogeneity of our study populations makes our study non-conclusive and this is a serious limitation. Children were not included into the control group, so those two groups were not completely age-matched and they differed in the lower quartile for age (47 and 13). Also, heatmap (Figure 1) clearly shows that the two newborns cluster to the controls and third newborn clusters to the meningitis group. Therefore, we cannot exclude possible age-dependent effects.

In addition, pathogen specific subgroup analysis is also complicated. In our study we mainly had patients with *S. pneumoniae* and very few patients infected with other pathogens. At the same time the “other pathogens” group was very heterogeneous. Therefore, results from pathogen comparison should be treated with caution.

Moreover, our analysis was based on the RNA extracted from the whole blood, not from the enriched Peripheral Blood Monocyte Cells (PBMCs) fraction. We acknowledge that the composition of the circulating blood cells in patients with BM and healthy controls is very different and that analysis of PBMCs would give a more focused description of the transcriptome. However, the whole blood may even be more advantageous as PBMCs reduce the number of neutrophils and in the case on BM, gene expression patterns from neutrophils may dominate the gene expression profile (Del Tordello et al., 2012; Li et al., 2012). Therefore, the analysis of the whole blood may present a more complete and systemic picture of changes in the blood transcriptome.

Our findings from the genome-wide expression profiling study indicate that there is a significant immune activation at the level of the transcriptome in patients with BM and we suggest that this could partly explain the complicated clinical course and poor outcome of BM. However, these findings are not conclusive due to the limited size and heterogeneity of our samples and should be verified in studies using a larger sample size and more homogeneous or matched population.

### Conflict of interest statement

The authors declare that the research was conducted in the absence of any commercial or financial relationships that could be construed as a potential conflict of interest.

## Appendix

**Table A1:** **Characteristics of the control group used in present study**.

	**Age**	**Sex**
K003	85	Female
K004	58	Female
K005	81	Male
K006	52	Male
K007	60	Female
K008	40	Female
K009	39	Female
K010	61	Female
K011	48	Female
K012	28	Male
K013	47	Female
K014	58	Female
K015	52	Female
K016	47	Female
K018	60	Female
K020	51	Female
K024	72	Male
K027	56	Male

**Table A2:** **The function of the most significantly up- and down-regulated genes described in Table 2**.

**Genes**	**Function**
FCER1A	This gene encodes the alpha subunit of immunoglobulin epsilon receptor (IgE receptor), what is the initiator of the allergic response. It is found on the surface of mast cells and basophils.
CPA3	Mast cell specific secretory granule metalloexopeptidase that regulates innate immune response. By degrading vasoactive peptides, CPA3 is protecting against sepsis and reduce mortality.
MS4A2(FCER1B)	Encodes the beta subunit of immunoglobulin epsilon receptor (IgE receptor), what is the initiator of the allergic response. It is found on the surface of mast cells and basophils.
DACH1	Encodes a chromatin-associated protein that associates with other DNA-binding transcription factors to regulate gene expression and cell fate determination during development.
HRB	The encoded protein binds the activation domain of the human immunodeficiency virus Rev protein when Rev is assembled onto its RNA target.
HDC	This gene encodes a enzyme that converts L-histidine to histamine. Histamine regulates several physiologic processes, including neurotransmission, gastric acid secretion, inflamation, and smooth muscle tone.
GRB10	This gene encodes a growth factor receptor-binding protein that interacts with insulin receptors and insulin-like growth-factor receptors.
PAG1	The protein encoded by this gene is involved in the regulation of T cell activation.
NR3C2	This gene encodes the mineralocorticoid receptor, which mediates aldosterone actions on salt and water balance within restricted target cells.
VNN1	This gene may play a role in oxidative-stress response.
MID2	The protein is member of ubiquitin-proteasome system.
IRAK3	This protein is primarily expressed in monocytes and macrophages and functions as a negative regulator of Toll-like receptor signaling and is associated with a susceptibility to asthma.
IPO11	Importins mediate nucleocytoplasmic transport of protein and RNA cargoes.
TMED8	Transmembrane emp24 protein transport domain containing 8.
ANKRD34B	A novel phosphoprotein is induced during bone marrow commitment to dendritic cells.
PDGFC	The protein encoded by this gene is a mitogenic factor for cells of mesenchymal origin.
IL18R1	This receptor specifically binds interleukin 18 (IL18), and is essential for IL18 mediated signal transduction, associated with asthma.

**Table A3:** **Function of genes from Table 3 (gene expression profile related to the type of pathogen)**.

**Genes**	**Function**
SEPHS1	This gene encodes an enzyme that synthesizes selenophosphate from selenide and ATP.
TRAF3IP3	The gene encodes a protein that mediates cell growth by modulating the c-Jun N-terminal kinase signal transduction pathway.
PIGW	Glycosylphosphatidylinositol (GPI) is a complex glycolipid that anchors many proteins to the cell surface.
FOXRED1	The encoded protein is localized to the mitochondria and may function as a chaperone protein required for the function of mitochondrial complex I.
WWC2-AS2	WWC2 antisense RNA 2.
RNU2-1	RNU2-1 forms spliceosome what catalyzes the removal of introns from nuclear mRNA precursors.
C14orf1	Chromosome 14 open reading frame 1 was identified as overexpressed on pancreatic cancer cell lines.
LAS1L	LAS1L interacts with the mammalian Rix1 complex to regulate ribosome biogenesis.
FHIT	This gene, a member of the histidine triad gene family is involved in purine metabolism.
NME1	This gene (NME1) was identified because of its reduced mRNA transcript levels in highly metastatic cells. It is biomarker of malignancy for some tumors.
TESK2	This gene product is a serine/threonine protein kinase what is involved in sprmatogenesis.
PSMA8	Proteasome subunit.
BYSL	Bystin may play multiple roles in mammalian cells, a conserved function is to facilitate ribosome biogenesis required for cell growth.
ZDHHC18	Palmitoyltransferase activity.
N4BP2	This protein binds and hydrolyzes ATP, may function as a 5'-polynucleotide kinase, and has the capacity to be a ubiquitylation substrate.
IPO4	Importins 4 and 7 accomplish nuclear import of HIF-1alpha more efficiently than the classical importin alpha/beta NLS receptor.
GPR18	Orphan G-protein-coupled receptor GPR18 with N-arachidonylglycine as the endogenous ligand.
TET1	Tet methylcytosine dioxygenase 1, nuclear exclusion of TET1 is associated with loss of 5-hydroxymethylcytosine in IDH1 wild-type gliomas.
SFXN2	Proteasome related gene, associated with genetic risk for Parkinson's disease.
CHI3L2	The encoded protein is secreted and is involved in cartilage biogenesis.
IKZF2	This gene is involved in the regulation of lymphocyte and in early hematopoietic development.

## References

[B1] Bleehen S. S. Thomas S. E. Greaves M. W. Newton J. Kennedy C. T. Hindley F. (1987). Cimetidine and chlorpheniramine in the treatment of chronic idiopathic urticaria: a multi-centre randomized double-blind study. Br. J. Dermatol. 117, 81–88 3307890 10.1111/j.1365-2133.1987.tb04095.x

[B2] Bone R. C. Balk R. A. Cerra F. B. Dellinger R. P. Fein A. M. Knaus W. A. (1992). Definitions for sepsis and organ failure and guidelines for the use of innovative therapies in sepsis. The ACCP/SCCM Consensus Conference Committee. American College of Chest Physicians/Society of Critical Care Medicine. Chest 101, 1644–1655 1303622 10.1378/chest.101.6.1644

[B3] Bruhn C. (2011). [Mast cells - more than allergy mediators. News from mast cell research]. Med. Monatsschr. Pharm. 34, 206–208 21812251

[B4] Chandrashekhar V. M. Halagali K. S. Nidavani R. B. Shalavadi M. H. Biradar B. S. Biswas D. (2011). Anti-allergic activity of German chamomile (*Matricaria recutita L.*) in mast cell mediated allergy model. J. Ethnopharmacol. 137, 336–340 10.1016/j.jep.2011.05.029 21651969

[B5] Chaudhuri A. Martinez-Martin P. Kennedy P. G. Andrew Seaton R. Portegies P. Bojar M. (2008). EFNS guideline on the management of community-acquired bacterial meningitis: report of an EFNS Task Force on acute bacterial meningitis in older children and adults. Eur. J. Neurol. 15, 649–659 10.1111/j.1468-1331.2008.02193.x 18582342

[B6] Coimbra R. S. Voisin V. De Saizieu A. B. Lindberg R. L. Wittwer M. Leppert D. (2006). Gene expression in cortex and hippocampus during acute pneumococcal meningitis. BMC Biol. 4:15 10.1186/1741-7007-4-15 16749930 PMC1523193

[B7] Deisenhammer F. Bartos A. Egg R. Gilhus N. E. Giovannoni G. Rauer S. (2006). Guidelines on routine cerebrospinal fluid analysis. Report from an EFNS task force. Eur. J. Neurol. 13, 913–922 10.1111/j.1468-1331.2006.01493.x 16930354

[B8] Del Tordello E. Bottini S. Muzzi A. Serruto D. (2012). Analysis of the regulated transcriptome of *Neisseria meningitidis* in human blood using a tiling array. J. Bacteriol. 194, 6217–6232 10.1128/JB.01055-12 22984255 PMC3486417

[B9] Echenique-Rivera H. Muzzi A. Del Tordello E. Seib K. L. Francois P. Rappuoli R. (2011). Transcriptome analysis of *Neisseria meningitidis* in human whole blood and mutagenesis studies identify virulence factors involved in blood survival. PLoS Pathog. 7:e1002027 10.1371/journal.ppat.1002027 21589640 PMC3088726

[B10] Edberg J. C. Yee A. M. Rakshit D. S. Chang D. J. Gokhale J. A. Indik Z. K. (1999). The cytoplasmic domain of human FcgammaRIa alters the functional properties of the FcgammaRI.gamma-chain receptor complex. J. Biol. Chem. 274, 30328–30333 10.1074/jbc.274.42.30328 10514529

[B11] Ernst L. K. Duchemin A. M. Miller K. L. Anderson C. L. (1998). Molecular characterization of six variant Fcgamma receptor class I (CD64) transcripts. Mol. Immunol. 35, 943–954 9881690 10.1016/s0161-5890(98)00079-0

[B12] Fassbender K. Schminke U. Ries S. Ragoschke A. Kischka U. Fatar M. (1997). Endothelial-derived adhesion molecules in bacterial meningitis: association to cytokine release and intrathecal leukocyte-recruitment. J. Neuroimmunol. 74, 130–134 9119965 10.1016/s0165-5728(96)00214-7

[B13] Goldstein S. M. Kaempfer C. E. Kealey J. T. Wintroub B. U. (1989). Human mast cell carboxypeptidase. Purification and characterization. J. Clin. Invest. 83, 1630–1636 10.1172/JCI114061 2708524 PMC303870

[B14] Grammatikos A. P. (2008). The genetic and environmental basis of atopic diseases. Ann. Med. 40, 482–495 10.1080/07853890802082096 18608118

[B15] Hotchkiss R. S. Karl I. E. (2003). The pathophysiology and treatment of sepsis. N. Engl. J. Med. 348, 138–150 10.1056/NEJMra021333 12519925

[B16] Koedel U. Angele B. Rupprecht T. Wagner H. Roggenkamp A. Pfister H. W. (2003). Toll-like receptor 2 participates in mediation of immune response in experimental pneumococcal meningitis. J. Immunol. 170, 438–444 12496429 10.4049/jimmunol.170.1.438

[B17] Konat G. W. Kielian T. Marriott I. (2006). The role of Toll-like receptors in CNS response to microbial challenge. J. Neurochem. 99, 1–12 10.1111/j.1471-4159.2006.04076.x 16899072 PMC2423668

[B18] Li P. Bai J. Li J. X. Zhang G. L. Song Y. H. Li Y. F. (2012). Molecular cloning, sequencing, and expression of the outer membrane protein P2 gene of *Haemophilus parasuis*. Res. Vet. Sci. 93, 736–742 10.1016/j.rvsc.2011.08.019 21945800

[B19] Majumder P. Gomez J. A. Boss J. M. (2006). The human major histocompatibility complex class II HLA-DRB1 and HLA-DQA1 genes are separated by a CTCF-binding enhancer-blocking element. J. Biol. Chem. 281, 18435–18443 10.1074/jbc.M601298200 16675454

[B20] Manikandan J. Kothandaraman N. Hande M. P. Pushparaj P. N. (2012). Deciphering the structure and function of FcepsilonRI/mast cell axis in the regulation of allergy and anaphylaxis: a functional genomics paradigm. Cell. Mol. Life Sci. 69, 1917–1929 10.1007/s00018-011-0886-0 22146792 PMC11114762

[B21] Mathias C. B. Freyschmidt E. J. Caplan B. Jones T. Poddighe D. Xing W. (2009). IgE influences the number and function of mature mast cells, but not progenitor recruitment in allergic pulmonary inflammation. J. Immunol. 182, 2416–2424 10.4049/jimmunol.0801569 19201896 PMC2653867

[B22] Natsuaki M. Stewart C. B. Vanderslice P. Schwartz L. B. Natsuaki M. Wintroub B. U. (1992). Human skin mast cell carboxypeptidase: functional characterization, cDNA cloning, and genealogy. J. Invest. Dermatol. 99, 138–145 1629626 10.1111/1523-1747.ep12616776

[B23] Nduka O. O. Parrillo J. E. (2009). The pathophysiology of septic shock. Crit. Care Clin. 25, 677–702 10.1016/j.ccc.2009.08.002 19892247

[B24] Neher J. J. Brown G. C. (2007). Neurodegeneration in models of Gram-positive bacterial infections of the central nervous system. Biochem. Soc. Trans. 35, 1166–1167 10.1042/BST0351166 17956303

[B25] Pathan N. Hemingway C. A. Alizadeh A. A. Stephens A. C. Boldrick J. C. Oragui E. E. (2004). Role of interleukin 6 in myocardial dysfunction of meningococcal septic shock. Lancet 363, 203–209 10.1016/S0140-6736(03)15326-3 14738793

[B26] Piliponsky A. M. Chen C. C. Nishimura T. Metz M. Rios E. J. Dobner P. R. (2008). Neurotensin increases mortality and mast cells reduce neurotensin levels in a mouse model of sepsis. Nat. Med. 14, 392–398 10.1038/nm1738 18376408 PMC2873870

[B27] Reijmerink N. E. Postma D. S. Bruinenberg M. Nolte I. M. Meyers D. A. Bleecker E. R. (2008). Association of IL1RL1, IL18R1, and IL18RAP gene cluster polymorphisms with asthma and atopy. J. Allergy Clin. Immunol. 122, 651–654 e658. 10.1016/j.jaci.2008.06.030 18774397

[B28] Rueff F. Friedl T. Arnold A. Kramer M. Przybilla B. (2011). Release of mast cell tryptase into saliva: a tool to diagnose food allergy by a mucosal challenge test? Int. Arch. Allergy Immunol. 155, 282–288 10.1159/000320492 21293148

[B29] Saito H. (2008). Progress in allergy signal research on mast cells: systemic approach to mast cell biology in allergic diseases. J. Pharmacol. Sci. 106, 341–346 18360090 10.1254/jphs.fm0070192

[B30] Sanak M. Potaczek D. P. Sznajd J. Musial J. Szczeklik A. (2009). Genetic associations of variants of the high affinity receptor for immunoglobulin E in Wegener's granulomatosis. Pol. Arch. Med. Wewn. 119, 170–174 19514647

[B31] Schubert-Unkmeir A. Slanina H. Frosch M. (2009). Mammalian cell transcriptome in response to meningitis-causing pathogens. Expert Rev. Mol. Diagn. 9, 833–842 10.1586/erm.09.68 19895228

[B32] Sehmi R. Wood L. J. Watson R. Foley R. Hamid Q. O'Byrne P. M. (1997). Allergen-induced increases in IL-5 receptor alpha-subunit expression on bone marrow-derived CD34+ cells from asthmatic subjects. A novel marker of progenitor cell commitment towards eosinophilic differentiation. J. Clin. Invest. 100, 2466–2475 10.1172/JCI119789 9366561 PMC508447

[B33] Smyth G. K. (2004). Linear models and empirical bayes methods for assessing differential expression in microarray experiments. Stat. Appl. Genet. Mol. Biol. 3:3 10.2202/1544-6115.1027 16646809

[B34] Stone R. (1994). Search for sepsis drugs goes on despite past failures. Science 264, 365–367 10.1126/science.8153620 8153620

[B35] Storey J. D. Tibshirani R. (2003). Statistical significance for genomewide studies. Proc. Natl. Acad. Sci. U.S.A. 100, 9440–9445 10.1073/pnas.1530509100 12883005 PMC170937

[B36] Trojano M. Avolio C. Simone I. L. Defazio G. Manzari C. De Robertis F. (1996). Soluble intercellular adhesion molecule-1 in serum and cerebrospinal fluid of clinically active relapsing-remitting multiple sclerosis: correlation with Gd-DTPA magnetic resonance imaging-enhancement and cerebrospinal fluid findings. Neurology 47, 1535–1541 8960741 10.1212/wnl.47.6.1535

[B37] Van Vugt M. J. Heijnen A. F. Capel P. J. Park S. Y. Ra C. Saito T. (1996). FcR gamma-chain is essential for both surface expression and function of human Fc gamma RI (CD64) *in vivo*. Blood 87, 3593–3599 8611682

[B38] Van Vugt M. J. Kleijmeer M. J. Keler T. Zeelenberg I. Van Dijk M. A. Leusen J. H. (1999). The FcgammaRIa (CD64) ligand binding chain triggers major histocompatibility complex class II antigen presentation independently of its associated FcR gamma-chain. Blood 94, 808–817 10397749

[B39] Warren H. S. (1997). Strategies for the treatment of sepsis. N. Engl. J. Med. 336, 952–953 10.1056/NEJM199703273361311 9070479

[B40] Weisfelt M. De Gans J. Van Der Poll T. Van De Beek D. (2006). Pneumococcal meningitis in adults: new approaches to management and prevention. Lancet Neurol. 5, 332–342 10.1016/S1474-4422(06)70409-4 16545750

